# Supplementary material for: Mid-life association between cardiovascular risk factors and cerebral blood flow in a multi-ethnic population
Source: Cereb Circ Cogn Behav. 2025 Apr 5;8:100384. doi: 10.1016/j.cccb.2025.100384 (PMC12008139; doi:10.1016/j.cccb.2025.100384)
Supplement: Supplementary file 1 [file mmc1.docx]

**Supplementary Table 1:** Characteristics of participants of the first CV risk factor visit with available data and all participants. BMI = body mass index. CBF = cerebral blood flow. CVD = cardiovascular disease. GFR = glomerular filtration rate. IQR = interquartile range. LDL = low density lipoprotein. SD = standard deviation. sCoV = spatial coefficient of variation.

|  | **Dataset for analyses**  *n = 531* | **All participants**  *n = 571* |
| --- | --- | --- |
| **Included in reference group**, n (%) | 281 (52.9) | 303 (53.1) |
| **Demographics** |  |  |
| Age (years), median [IQR] | 53.0 [47.0, 58.0] | 52.0 [47.0, 58.0] |
| Sex (female), n (%) | 238 (44.8) | 257 (45.0) |
| **BMI (kg/m^2^),** mean (SD) | 26.1 (4.0) | 26.1 (4.1) |
| **Blood pressure** |  |  |
| SBP (mmHg), mean (SD) | 129.7 (17.0) | 129.8 (17.1) |
| DBP (mmHg), mean (SD) | 80.8 (9.7) | 80.8 (9.8) |
| Hypertension, n (%) | 184 (34.9) | 206 (36.4) |
| **Smoking (yes)**, n (%) | 112 (21.3) | 121 (21.4) |
| **Diabetes mellitus**, n (%) | 53 (10.1) | 57 (10.1) |
| **Lipid profile** |  |  |
| Total cholesterol level (mmol/l), mean (SD) | 5.3 (1.1) | 5.3 (1.1) |
| LDL (mmol/l), mean (SD) | 3.3 (1.0) | 3.3 (1.0) |
| Lipid lowering medication, n (%) | 83 (15.7) | 91 (16.1) |
| **Estimated GFR (mL/min/1.73 m^2^)**, mean (SD) | 97.1 (13.3) | 96.9 (13.4) |
| **Haematocrit (%)**, mean (SD) | 44.0 (3.8) | 43.9 (3.8) |
| **History of CVD,** n (%) | 29 (5.5) | 32 ( 5.7) |

**Supplementary Table 2:** Full models of the association of demographics and first and second CV risk factor visit with CBF (A) and standardized log-transformed sCoV (B) as derived from multivariate linear regression analyses. All models were multivariate models, adjusted for age, sex, ethnicity, follow-up time, BMI, smoking, diabetes mellitus, hypertension, total cholesterol levels, eGFR levels, and a history of CVD. Inverse probability weighting was used to correct for study design. Estimates for follow-up time were omitted from this table. * Statistically significant (P<0.05). BMI = body mass index. CI = confidence interval. CVD = cardiovascular diseases. DBP = diastolic blood pressure. eGFR = estimated glomerular filtration rate. SBP = systolic blood pressure. sCoV = spatial coefficient of variation.

| **A** | **First CV risk factor visit** | | **Second CV risk factor visit** | |
| --- | --- | --- | --- | --- |
|  | GM CBF  (mL/min/100g) | WM CBF  (mLmin/100g) | GM CBF  (mL/min/100g) | WM CBF  (mL/min/100g) |
|  | *Estimate (95% CI)* | *Estimate (95% CI)* | *Estimate (95% CI)* | *Estimate (95% CI)* |
| **Demographics** |  |  |  |  |
| Age (years) | -0.23 (-0.34 ; -0.11)* | 0.03 (-0.02 ; 0.07) | -0.22 (-0.34 ; -0.1)* | 0.03 (-0.02 ; 0.07) |
| Sex (female) | -0.43 (-2.05 ; 1.18) | 0.54 (-0.08 ; 1.16) | -0.65 (-2.27 ; 0.96) | 0.59 (-0.03 ; 1.2) |
| Ethnicity (reference = Dutch) |  |  |  |  |
| South-Asian Surinamese | -1.46 (-3.25 ; 0.33) | -1.59 (-2.28 ; -0.9)* | -1.63 (-3.44 ; 0.19) | -1.57 (-2.26 ; -0.88)* |
| Moroccan | -2.45 (-5.07 ; 0.16) | -1.15 (-2.15 ; -0.14)* | -2.91 (-5.43 ; -0.39)* | -1.36 (-2.31 ; -0.4)* |
|  |  |  |  |  |
| **CV risk factor** |  |  |  |  |
| BMI (kg/m^2^) | 0.13 (-0.08 ; 0.35) | 0.01 (-0.07 ; 0.09) | 0.12 (-0.09 ; 0.33) | -0.02 (-0.1 ; 0.06) |
| Smoking (yes) | 1.21 (-0.6 ; 3.02) | -0.02 (-0.71 ; 0.68) | 1.07 (-0.97 ; 3.1) | -0.67 (-1.45 ; 0.1) |
| Diabetes mellitus (yes) | 2.45 (-0.25 ; 5.16) | 0.07 (-0.97 ; 1.11) | 1.45 (-0.87 ; 3.78) | -0.24 (-1.12 ; 0.64) |
| SBP (mmHg) | 0.03 (-0.04 ; 0.11) | 0.01 (-0.02 ; 0.03) | 0.04 (-0.03 ; 0.1) | 0.02 (0 ; 0.05) |
| DBP (mmHg) | -0.05 (-0.18 ; 0.07) | 0 (-0.05 ; 0.04) | -0.1 (-0.22 ; 0.02) | -0.03 (-0.08 ; 0.01) |
| Total cholesterol (mmol/l) | -0.15 (-0.9 ; 0.6) | -0.14 (-0.43 ; 0.15) | -0.37 (-1.03 ; 0.29) | -0.35 (-0.6 ; -0.09)* |
| eGFR (mL/min/1.73 m²) | -0.02 (-0.09 ; 0.04) | -0.01 (-0.03 ; 0.02) | 0.01 (-0.05 ; 0.07) | 0.02 (-0.01 ; 0.04) |
| History of CVD (yes) | -3.34 (-6.46 ; -0.22)* | -0.65 (-1.85 ; 0.54) | -4.06 (-6.62 ; -1.51)* | -0.99 (-1.96 ; -0.02)* |

| **B** | **First CV risk factor visit** | | **Second CV risk factor visit** | |
| --- | --- | --- | --- | --- |
|  | GM sCoV (z-score) | WM sCoV (z-score) | GM sCoV (z-score) | WM sCoV (z-score) |
|  | *Estimate (95% CI)* | *Estimate (95% CI)* | *Estimate (95% CI)* | *Estimate (95% CI)* |
| **Demographics** |  |  |  |  |
| Age (years) | 0.02 (0.01 ; 0.04)* | 0.01 (-0.01 ; 0.02) | 0.03 (0.01 ; 0.04)* | 0.01 (-0.01 ; 0.02) |
| Sex (female) | -0.7 (-0.88 ; -0.51)* | -0.46 (-0.65 ; -0.27)* | -0.68 (-0.87 ; -0.5)* | -0.47 (-0.66 ; -0.29)* |
| Ethnicity (reference = Dutch) |  |  |  |  |
| South-Asian Surinamese | -0.07 (-0.27 ; 0.14) | 0.4 (0.2 ; 0.61)* | -0.08 (-0.28 ; 0.12) | 0.37 (0.17 ; 0.58)* |
| Moroccan | -0.37 (-0.66 ; -0.07)* | 0.1 (-0.2 ; 0.41) | -0.26 (-0.54 ; 0.03) | 0.24 (-0.04 ; 0.53) |
|  |  |  |  |  |
| **CV risk factors** |  |  |  |  |
| BMI (kg/m^2^) | 0.01 (-0.01 ; 0.03) | 0.02 (-0.01 ; 0.04) | 0.01 (-0.01 ; 0.04) | 0.03 (0 ; 0.05)* |
| Smoking (yes) | -0.16 (-0.36 ; 0.04) | 0.04 (-0.17 ; 0.25) | -0.04 (-0.27 ; 0.19) | 0.38 (0.15 ; 0.61)* |
| Diabetes mellitus (yes) | -0.13 (-0.43 ; 0.18) | 0.02 (-0.3 ; 0.33) | -0.03 (-0.29 ; 0.23) | 0.05 (-0.21 ; 0.32) |
| SBP (mmHg) | -0.01 (-0.02 ; 0)* | -0.01 (-0.01 ; 0) | -0.01 (-0.02 ; -0.01)* | -0.01 (-0.02 ; 0)* |
| DBP (mmHg) | 0.01 (0 ; 0.03) | 0.01 (-0.01 ; 0.02) | 0.02 (0.01 ; 0.03)* | 0.01 (0 ; 0.03)* |
| Total cholesterol (mmol/l) | -0.02 (-0.11 ; 0.06) | 0.05 (-0.04 ; 0.14) | 0.01 (-0.06 ; 0.09) | 0.08 (0.01 ; 0.16)* |
| eGFR (mL/min/1.73 m²) | 0 (-0.01 ; 0) | 0 (0 ; 0.01) | -0.01 (-0.01 ; 0) | 0 (-0.01 ; 0) |
| History of CVD (yes) | 0.08 (-0.28 ; 0.43) | 0.32 (-0.04 ; 0.69) | 0.3 (0.02 ; 0.59)* | 0.42 (0.13 ; 0.72)* |

| **Association** | | **Overall** | **Dutch**  n = 236 | **Moroccan**  n = 122 | **SA Surinamese**  n = 173 |
| --- | --- | --- | --- | --- | --- |
|  |  | *Estimate (95% CI)* | *Estimate (95% CI)* | *Estimate (95% CI)* | *Estimate (95% CI)* |
| **Outcome measure** | **CV risk factor** |  |  |  |  |
| CBF (ml/min/100g) |  |  |  |  |  |
| GM CBF | History of CVD | -3.16 (-5.63 ; -0.68)* | -3.54 (-7.58 ; 0.51) | -5.01 (-14.24 ; 4.23) | -3.22 (-6.73 ; 0.3) |
| WM CBF | Total cholesterol | -0.28 (-0.51 ; -0.04)* | -0.53 (-1.43 ; 0.38) | 0.13 (-1.48 ; 1.73) | -0.31 (-1.41 ; 0.79) |
| WM CBF | WML | 0.43 (0.09 ; 0.76)* | 0.63 (-0.68 ; 1.93) | 1.08 (-3.2 ; 1.05) | -0.02 (-1.59 ; 1.56) |
|  |  |  |  |  |  |
| sCoV (z-score) |  |  |  |  |  |
| GM sCoV | SBP | -0.01 (-0.01 ; 0)* | -0.01 (-0.01 ; 0) | -0.01 (-0.02 ; 0) | 0 (-0.01 ; 0) |
| WM sCoV | Smoking | 0.38 (0.15 ; 0.61)* | 0.59 (0.22 ; 0.96)* | 0.03 (-0.59 ; 0.66) | 0.21 (-0.19 ; 0.61) |
| WM sCoV | History of CVD | 0.38 (0.1 ; 0.67)* | 0.52 (0.04 ; 1)* | 1.19 (0.08 ; 2.3)* | 0.05 (-0.05 ; 0.16) |

**Supplementary Table 3:** Effect of ethnicity on the observed associations between CBF or standardized log-transformed sCoV and the CV risk factors measured at the second visit. Estimates represent the results from the risk-factor specific models. Estimates for the different ethnicities were derived from subgroup analyses. Risk factor-specific models were adjusted for age, sex, ethnicity, and follow-up time between the CV risk factor visits and MRI measurements. * Statistically significant (P<0.05). CBF = cerebral blood flow. CI = confidence interval. CVD = cardiovascular diseases. GM = grey matter. SA = South-Asian Surinamese. SBP = systolic blood pressure. sCoV = spatial coefficient of variation. WM = white matter. WML = white matter lesions.

**Supplementary Table 4:** Sensitivity analysis on the effect of partial volume correction (PVC) on the association between second CV risk factor visit and grey matter CBF and standardized log-transformed sCoV as derived from multivariate linear regression analyses. All models were multivariate models, adjusted for age, sex, ethnicity, follow-up time, BMI, smoking, diabetes mellitus, hypertension, total cholesterol levels, eGFR levels, and a history of CVD. Inverse probability weighting was used to correct for study design. Estimates for follow-up time were omitted from this table. * Statistically significant (P<0.05). BMI = body mass index. CBF = cerebral blood flow. CI = confidence interval. CVD = cardiovascular diseases. DBP = diastolic blood pressure. eGFR = estimated glomerular filtration rate. PVC = partial volume correction. SBP = systolic blood pressure.

|  | **Without PVC** | **With PVC** |
| --- | --- | --- |
|  | *Estimate (95% CI)* | *Estimate (95% CI)* |
| **CBF (ml/min/100g)** |  |  |
| CV risk factors |  |  |
| BMI (kg/m^2^) | 0.06 (-0.1 ; 0.22) | 0.12 (-0.09 ; 0.33) |
| Smoking (yes) | 0.7 (-0.87 ; 2.27) | 1.07 (-0.97 ; 3.1) |
| Diabetes mellitus (yes) | 0.48 (-1.31 ; 2.27) | 1.45 (-0.87 ; 3.78) |
| SBP (mmHg) | 0.04 (-0.01 ; 0.09) | 0.04 (-0.03 ; 0.1) |
| DBP (mmHg) | -0.08 (-0.18 ; 0.01) | -0.1 (-0.22 ; 0.02) |
| Total cholesterol (mmol/l) | -0.34 (-0.85 ; 0.17) | -0.37 (-1.03 ; 0.29) |
| eGFR (mL/min/1.73 m²) | 0.01 (-0.03 ; 0.06) | 0.01 (-0.05 ; 0.07) |
| History of CVD (yes) | -3.4 (-5.37 ; -1.42)* | -4.06 (-6.62 ; -1.51)* |
|  |  |  |
| **sCoV (z-score)** |  |  |
| CV risk factors |  |  |
| BMI (kg/m^2^) | 0.01 (-0.01 ; 0.04) | 0.01 (-0.01 ; 0.04) |
| Smoking (yes) | -0.04 (-0.27 ; 0.19) | -0.01 (-0.25 ; 0.23) |
| Diabetes mellitus (yes) | -0.03 (-0.29 ; 0.23) | 0 (-0.27 ; 0.28) |
| SBP (mmHg) | -0.01 (-0.02 ; -0.01)* | -0.01 (-0.02 ; -0.01)* |
| DBP (mmHg) | 0.02 (0.01 ; 0.03)* | 0.02 (0.01 ; 0.03)* |
| Total cholesterol (mmol/l) | 0.01 (-0.06 ; 0.09) | 0.02 (-0.06 ; 0.1) |
| eGFR (mL/min/1.73 m²) | -0.01 (-0.01 ; 0) | -0.01 (-0.01 ; 0)* |
| History of CVD (yes) | 0.3 (0.02 ; 0.59)* | 0.21 (-0.09 ; 0.51) |

|  | **Without correction for haematocrit** | **With correction for haematocrit** |
| --- | --- | --- |
|  | *Estimate (95% CI)* | *Estimate (95% CI)* |
| **CV risk factors** |  |  |
| BMI (kg/m^2^) | 0.01 (-0.17 ; 0.2) | 0.12 (-0.09 ; 0.33) |
| Smoking (yes) | 0.23 (-1.61 ; 2.06) | 1.07 (-0.97 ; 3.1) |
| Diabetes mellitus (yes) | 0.62 (-1.47 ; 2.71) | 1.45 (-0.87 ; 3.78) |
| SBP (mmHg) | 0.05 (-0.01 ; 0.1) | 0.04 (-0.03 ; 0.1) |
| DBP (mmHg) | -0.12 (-0.23 ; -0.02)* | -0.1 (-0.22 ; 0.02) |
| Total cholesterol (mmol/l) | -0.62 (-1.21 ; -0.02)* | -0.37 (-1.03 ; 0.29) |
| eGFR (mL/min/1.73 m²) | 0.02 (-0.03 ; 0.08) | 0.01 (-0.05 ; 0.07) |
| History of CVD (yes) | -4.4 (-6.69 ; -2.11)* | -4.06 (-6.62 ; -1.51)* |

**Supplementary Table 5:** Sensitivity analysis on the effect of haematocrit correction on the association between second CV risk factor visits and grey matter CBF, as derived from multivariate linear regression analyses. All models were multivariate models, adjusted for age, sex, ethnicity, follow-up time, BMI, smoking, diabetes mellitus, hypertension, total cholesterol levels, eGFR levels, and a history of CVD. Inverse probability weighting was used to correct for study design. Estimates for follow-up time were omitted from this table. * Statistically significant (P<0.05), adjusted for multiple comparisons using FDR correction. BMI = body mass index. CBF = cerebral blood flow. CI = confidence interval. CVD = cardiovascular diseases. DBP = diastolic blood pressure. eGFR = estimated glomerular filtration rate. SBP = systolic blood pressure.

**Supplementary Table 6:** Sensitivity analysis on the effect of exclusion of participants with a positive history of CVD at the second visit (n = 46) on the association between CV risk factors of the second visit and grey matter CBF, as derived from linear regression analyses. All models were multivariate models, adjusted for age, sex, ethnicity, follow-up time, BMI, smoking, diabetes mellitus, hypertension, total cholesterol levels, eGFR levels, and a history of CVD. Inverse probability weighting was used to correct for study design. Estimates for follow-up time were omitted from this table. * Statistically significant (P<0.050). BMI = body mass index. CBF = cerebral blood flow. CI = confidence interval. CVD = cardiovascular diseases. DBP = diastolic blood pressure. eGFR = estimated glomerular filtration rate. SBP = systolic blood pressure.

|  | **Exclusion of participants with a history of CVD (n = 485)** | **All participants (n = 531)** |
| --- | --- | --- |
|  | *Estimate (95% CI)* | *Estimate (95% CI)* |
| **CV risk factors** |  |  |
| BMI (kg/m^2^) | 0.11 (-0.11 ; 0.33) | 0.12 (-0.09 ; 0.33) |
| Smoking (yes) | 1.22 (-0.96 ; 3.39) | 1.07 (-0.97 ; 3.1) |
| Diabetes mellitus (yes) | 1.46 (-1.12 ; 4.04) | 1.45 (-0.87 ; 3.78) |
| SBP (mmHg) | 0.05 (-0.02 ; 0.13) | 0.04 (-0.03 ; 0.1) |
| DBP (mmHg) | -0.14 (-0.26 ; -0.01)* | -0.1 (-0.22 ; 0.02) |
| Total cholesterol (mmol/l) | -0.37 (-1.08 ; 0.34) | -0.37 (-1.03 ; 0.29) |
| eGFR (mL/min/1.73 m²) | 0.01 (-0.06 ; 0.07) | 0.01 (-0.05 ; 0.07) |
| History of CVD (yes) | - | -4.06 (-6.62 ; -1.51)* |


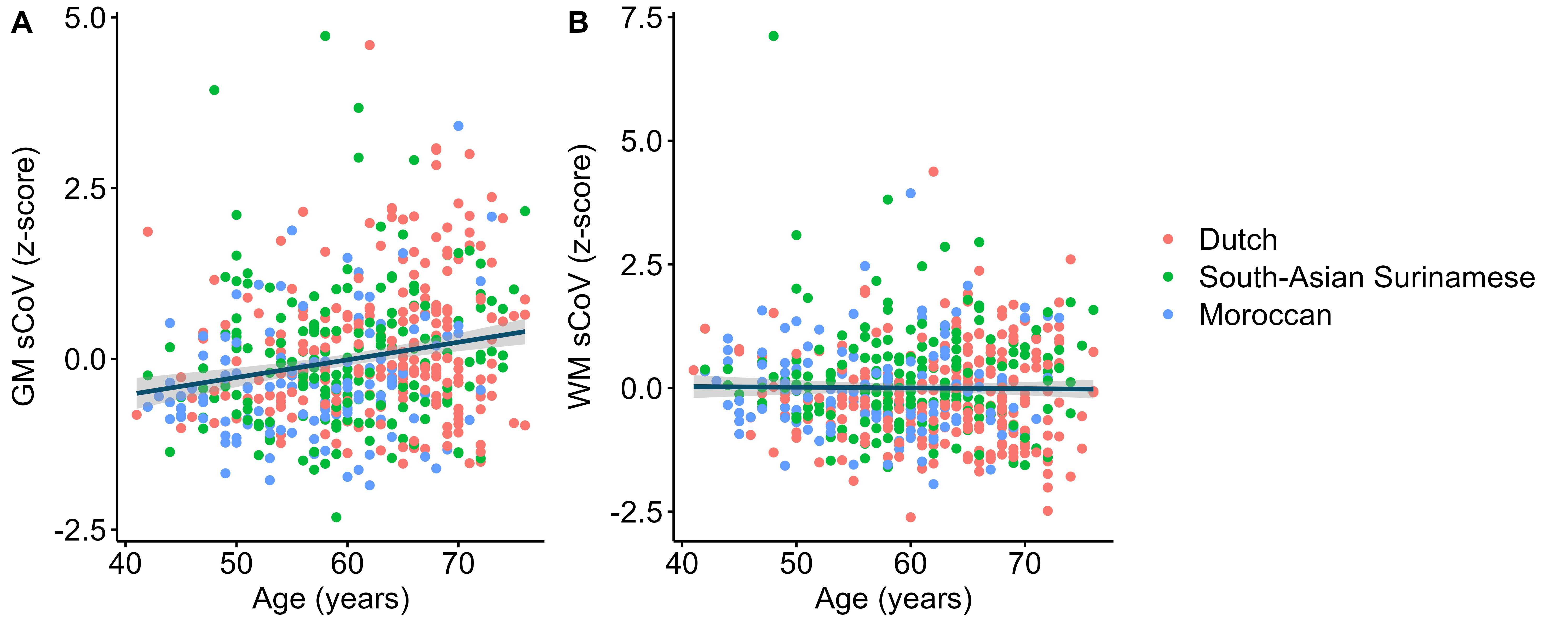


**Supplementary Figure 1:** Cross-sectional association between standardized log-transformed sCoV and age in grey matter (A) and white matter (B), stratified by ethnicity. GM = grey matter. sCoV = spatial coefficient of variation. WM = white matter.
